# Supplementary material for: Altitude as a key environmental factor shaping microbial communities of tea green leafhoppers (Matsumurasca onukii)
Source: Microbiol Spectr. 2023 Nov 3;11(6):e01009-23. doi: 10.1128/spectrum.01009-23 (PMC10714740; doi:10.1128/spectrum.01009-23)
Supplement: Supplemental Figures — Figures S1 to S9. [file spectrum.01009-23-s0001.docx]

**Altitude as a key environmental factor shaping microbial communities of tea green leafhoppers (*Matsumurasca onukii*)**

**Yong Zhang^a,b,c#^**, **Song Liu^a#^**, **Xue-yu Huang^a#^**, **Hua-bin Zi^a^**, **Tian Gao^a^**, **Rui-jie Ji^a^**, **Juan Sheng^a^**, **Dian Zhi^a^**, **Ying-lao Zhang^c*^, Chun-mei Gong^b*^ and Yun-qiu Yang^a*^**

^a^State Key Laboratory of Tea Plant Biology and Utilization, Anhui Agricultural University, Hefei, Anhui, China

^b^College of Horticulture, Northwest A＆F University, Yangling, Shaanxi, China

^c^College of Life Science, Anhui Agricultural University, Hefei, Anhui, China

^#^Yong Zhang, Song Liu and Xueyu Huang contributed equally to this work and share first authorship

^*^*Correspondence to: Yun-qiu Yang Email: [longyanhua@ahau.edu.cn；](mailto:yyq_lyh@anau.edu.cn;)Chun-mei Gong Email: [gcm228@nwafu.edu.cn](mailto:gcm228@nwafu.edu.cn); Yinglao Zhang Email: Zhangyl@ahau.edu.cn

**
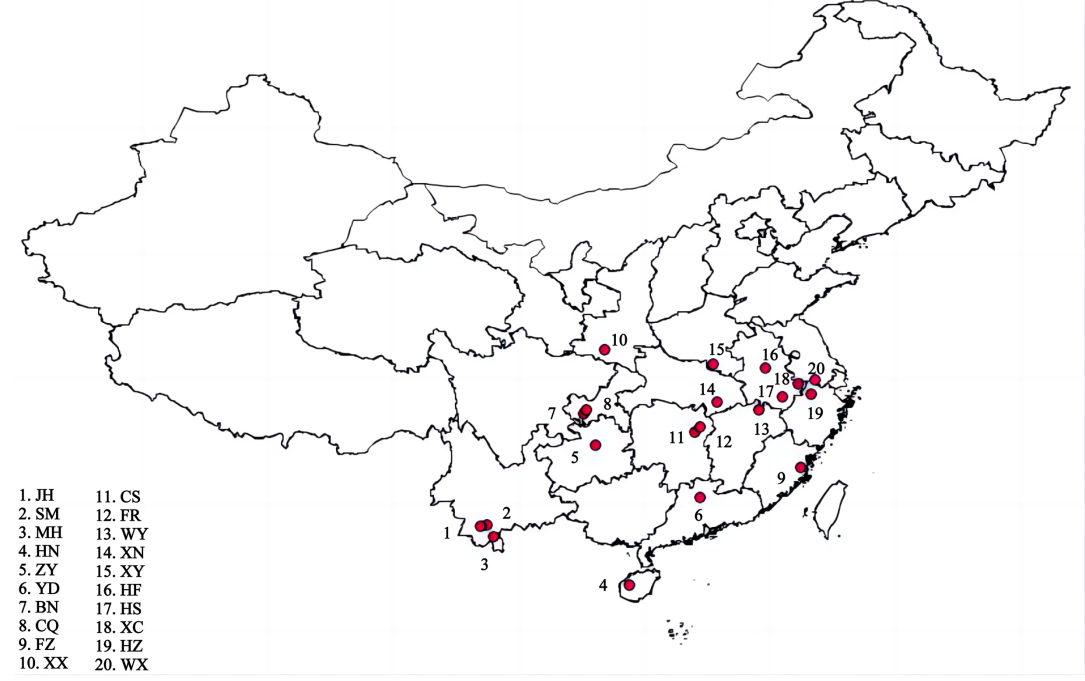
**

**FIG. S1 Collections sites of *M*. *onukii* in China**

Details related to the sites and dates of collection are provided in Table S1


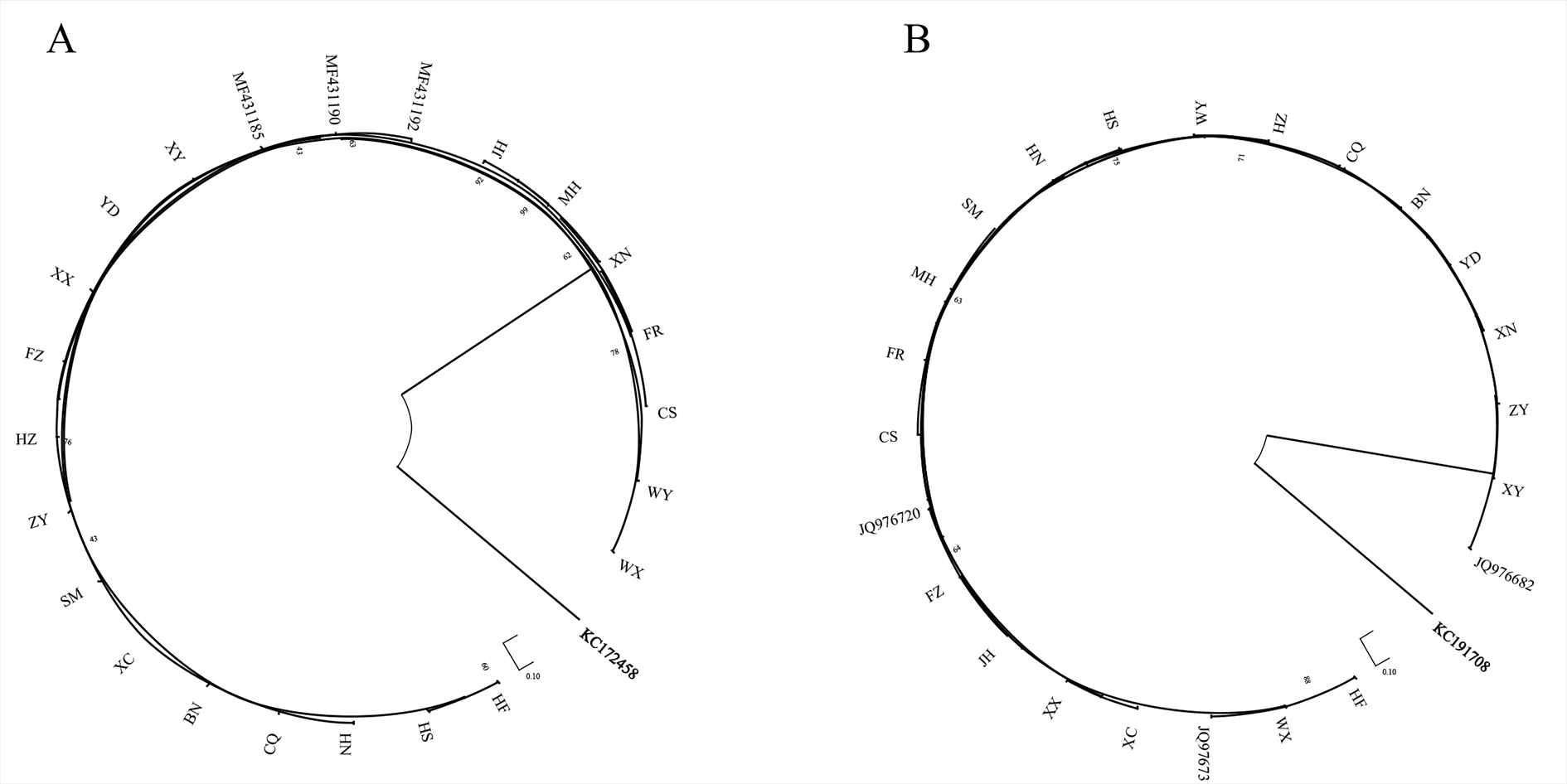


**FIG. S2 Maximum-likelihood (ML) phylogenetic tree constructed based on 16sRNA (B) gene fragment sequence and *COI* gene fragment (A) sequence of mitochondrial genome of *M*. *onukii*** Bar represents the amino acid substitution at each site. The bootstrap value is displayed on each node. MF431185, MF431192 and MF431190 are the 16sRNA gene sequence of the reference *M*. *onukii*, and KQ72458 is the 16S rRNA gene sequence of the out group *Emposca flavescens*; JQ976882, JQ976720 and JQ976731 are the COI gene sequences of *Empoasca onukii*, and KC191708 is the *COI* gene sequence of out group *Emposca flavescens*

**
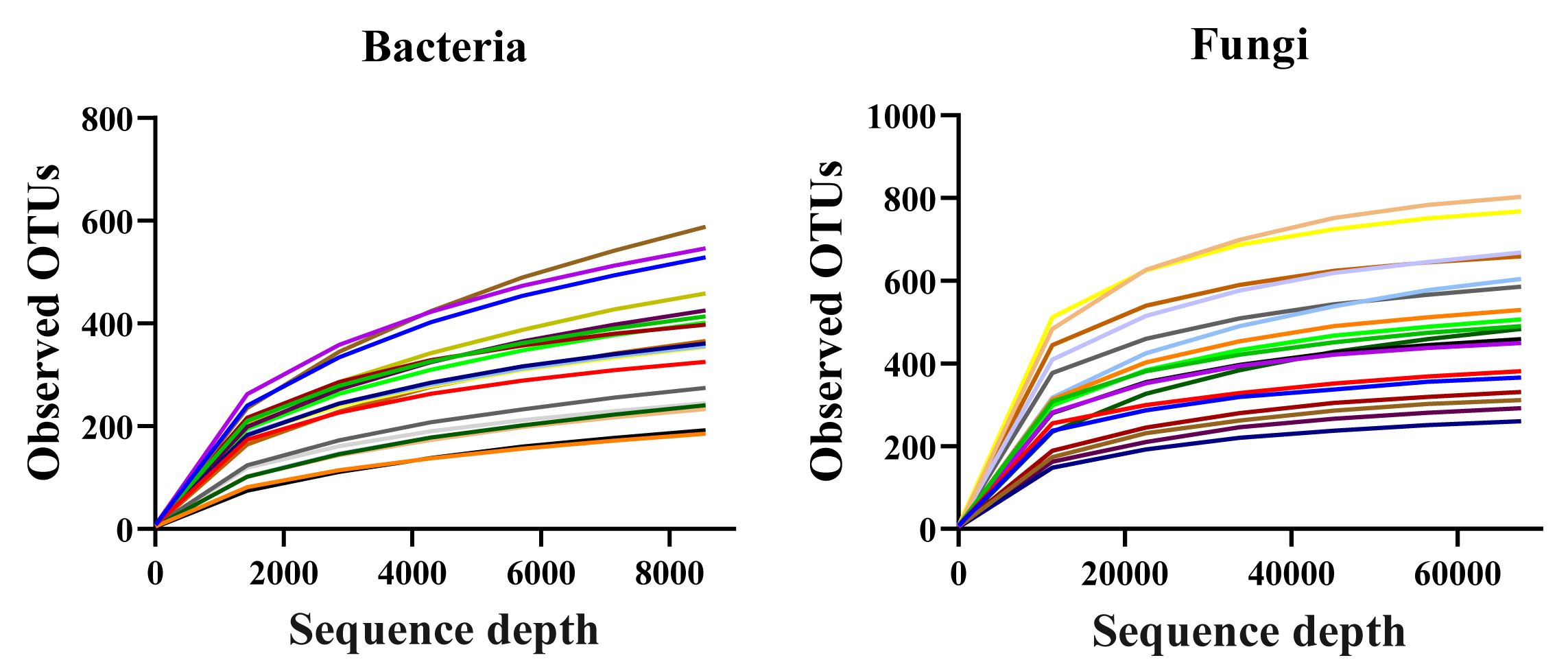
**

**FIG. S3 Rarefaction curves, based on number of operational taxonmic units (OTUs) observed of the bacterial and fungal communities**

**
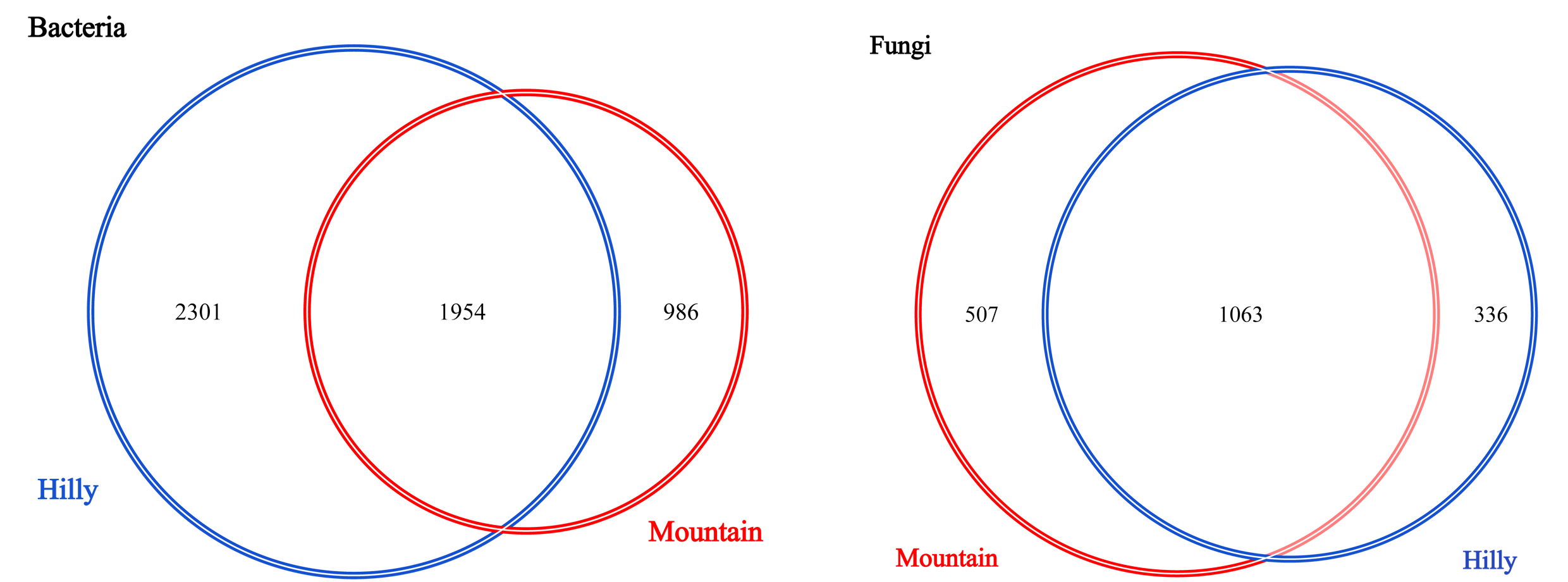
**

**FIG. S4 Venn diagram analysis showing number of shared operational taxonmic units (OTUs) among sample groups**

Hilly: samples collected from hilly area tea plantations; Mountain: samples collected from mountainous region tea plantations

**
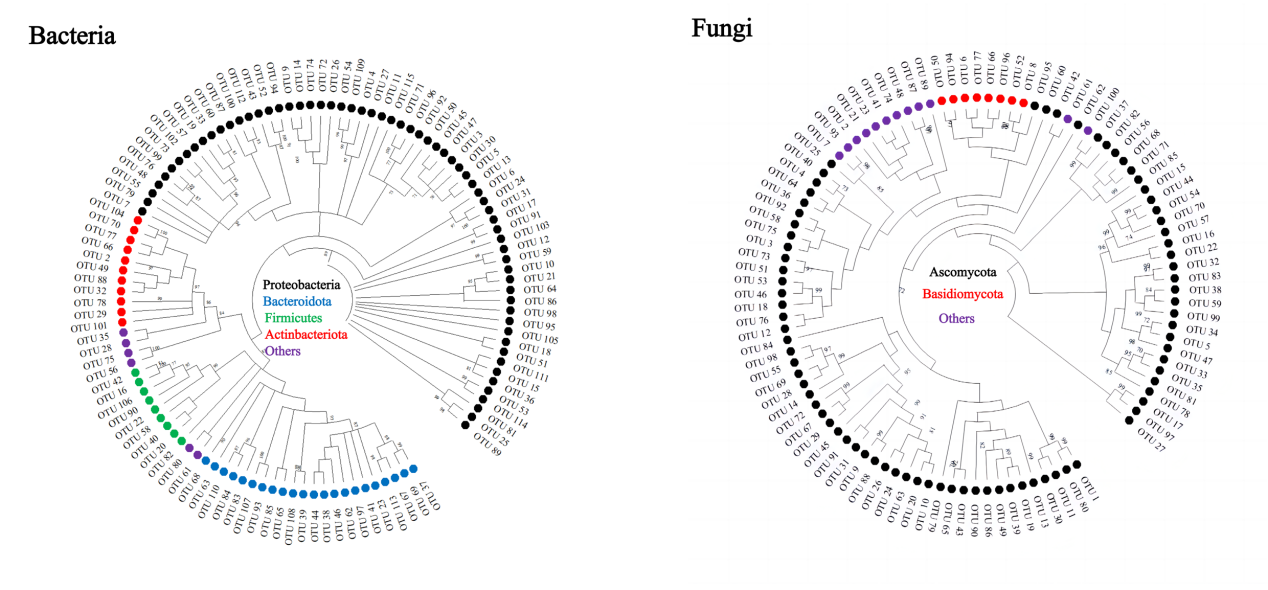
**

**FIG. S5 Maximum-likelihood (ML) phylogenetic tree constructed based on** **high abundance operational taxonmic units (OTUs) (top 10% in terms of relative abundance)**

**
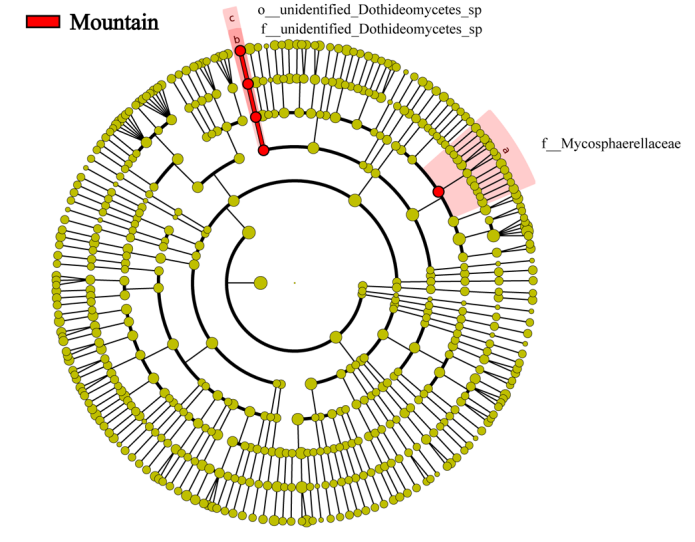
**

**FIG. S6 Differential abundance of fungal taxa associated with *M*. *onukii* as determined by Linear discriminant analysis Effect Size (LEfSe)**

Mountain: samples collected from mountainous region tea plantations

**
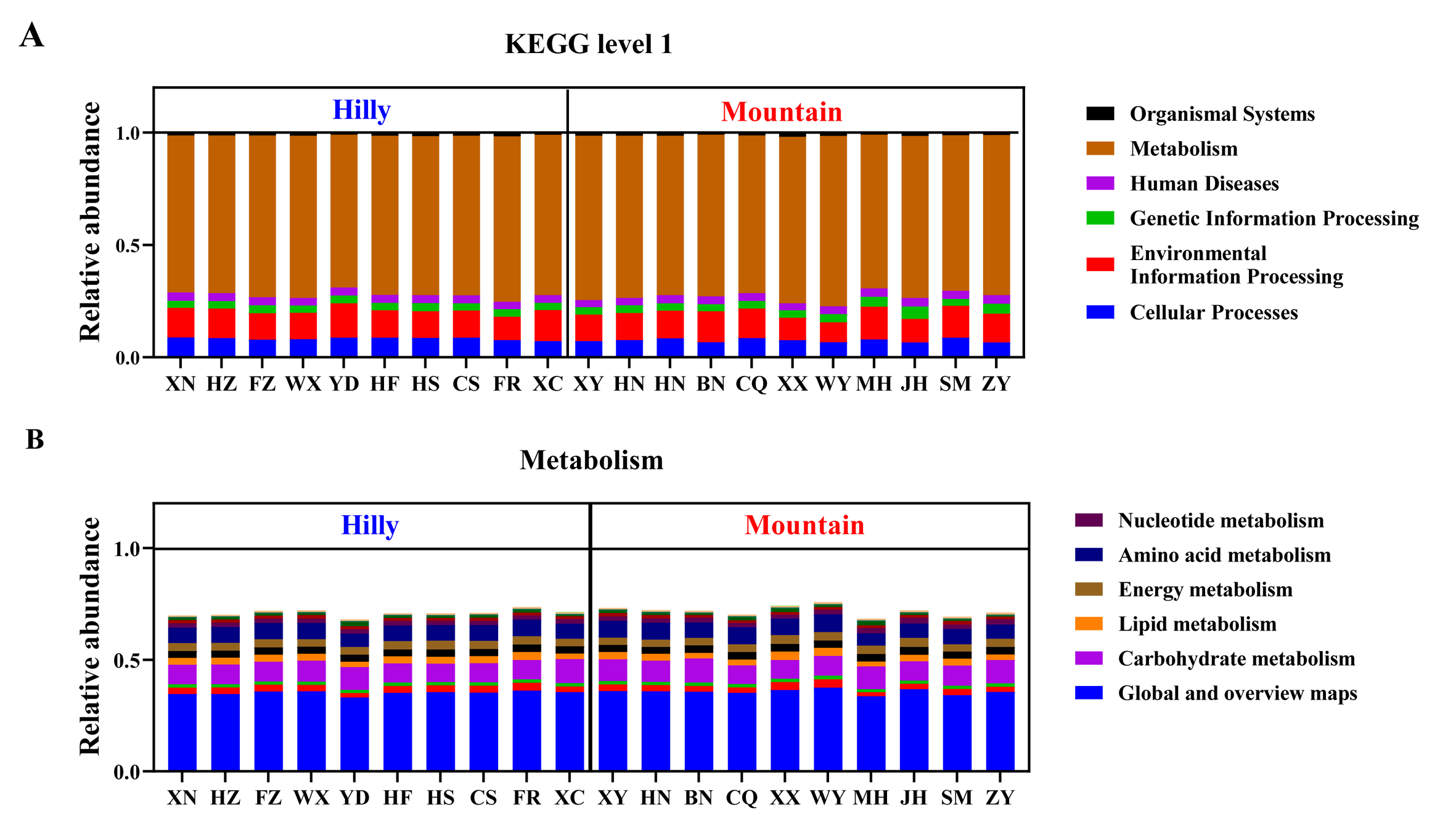
**

**FIG. S7 Relative abundance of predicted functional bacterial community associated with *M*. *onukii* as determined by Tax4Fun2**

The annotation results of the bacterial community of *M*. *onukii* at Kyoto Encyclopedia of Genes and Genomes (KEGG) level 1 (A); The annotation results of the bacterial community of *M*. *onukii* at Metabolism (belonging to Level 2). Hilly: samples collected from hilly area tea plantations; Mountain: samples collected from mountainous region tea plantations\

**
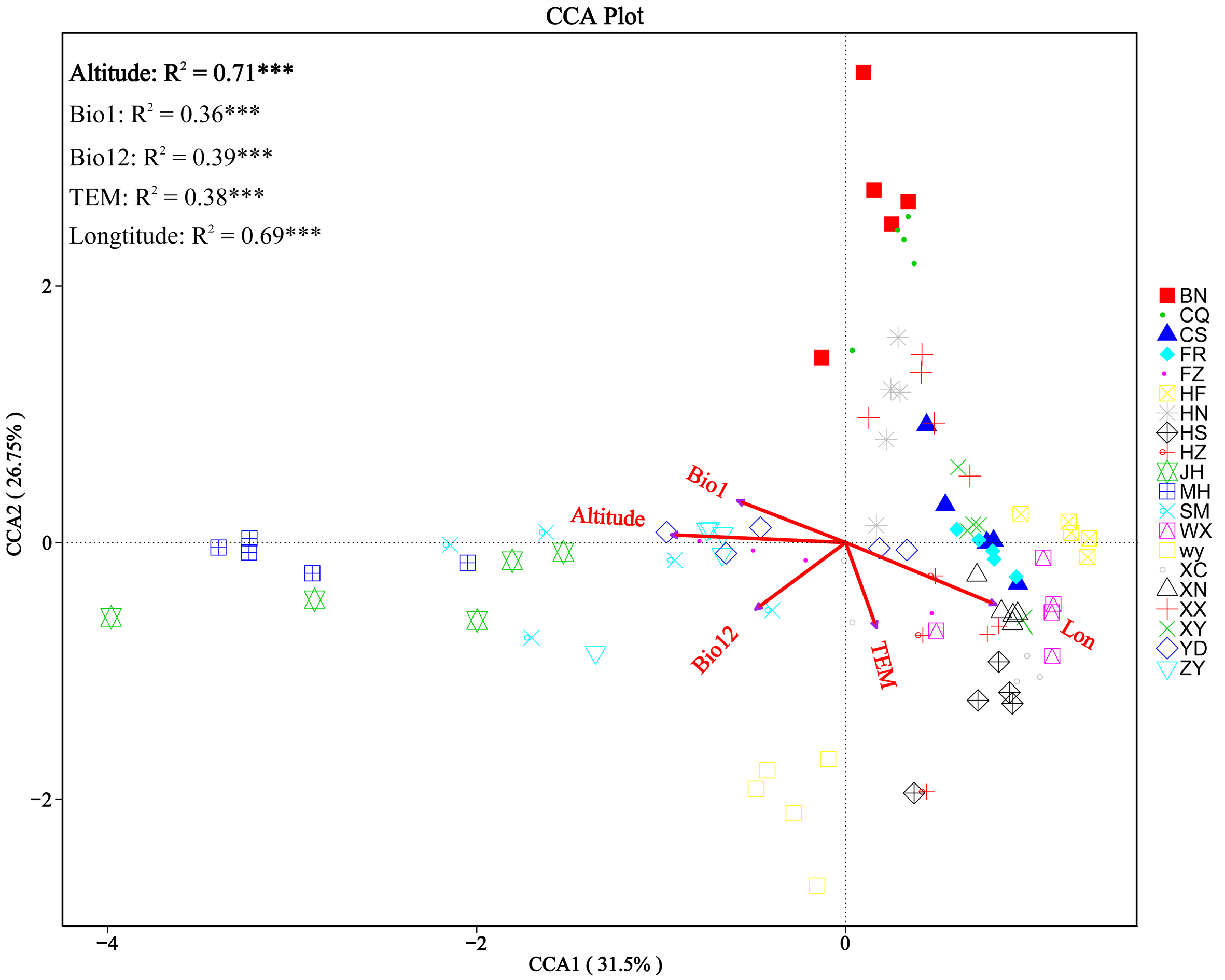
**

**FIG. S8 Relationship of the bacterial community copmosition associated with *M*. *onukii* with the major environmental factors revealed by c**anonical correspondence analysis (**CCA) at operational taxonmic units (OTUs) level**

Significance levels are denoted as follows: *^***^P* < 0.0001

**
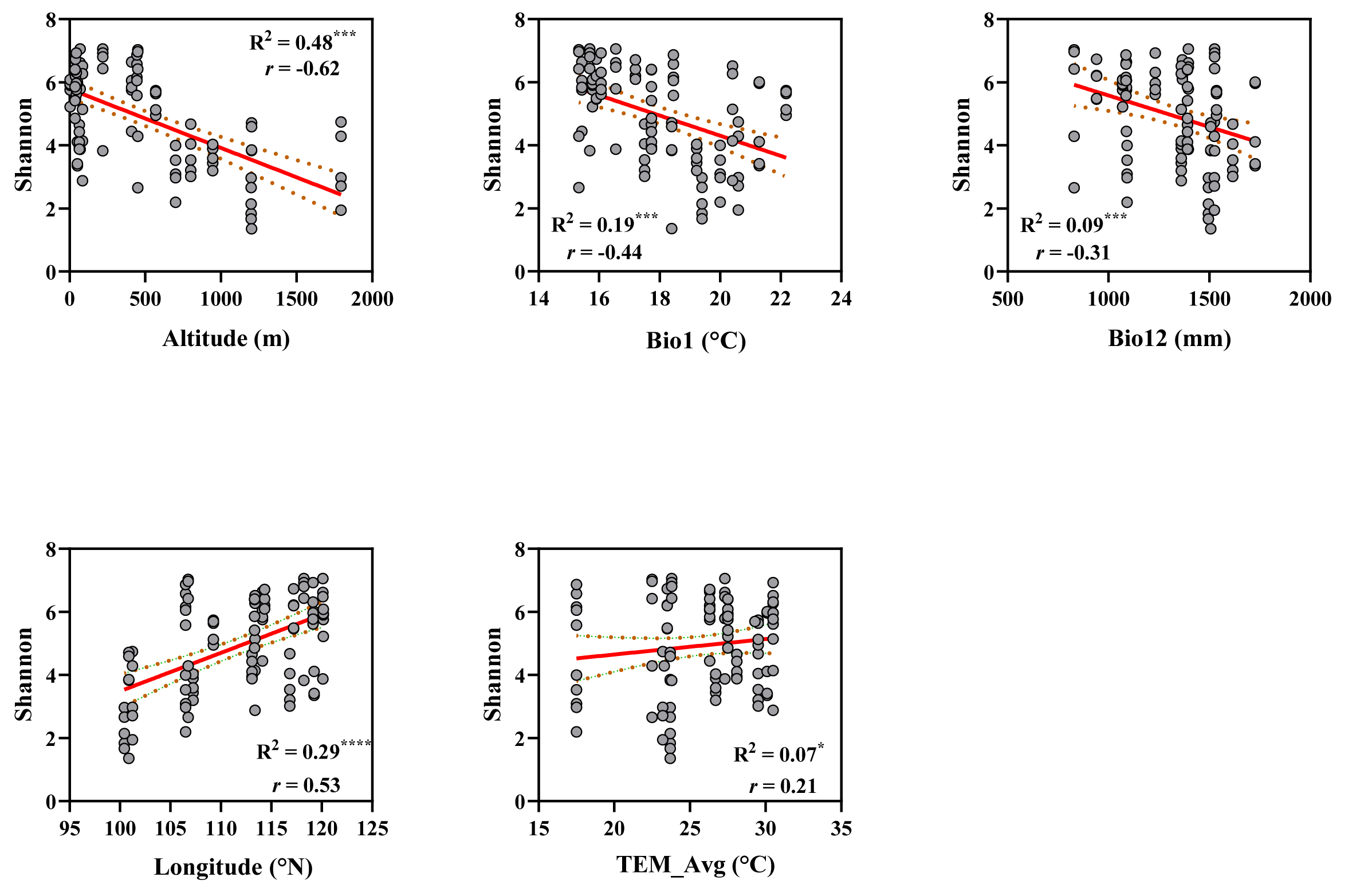
**

**FIG. S9 Relationships between bacterial α‐diversity and the main drivers were estimated via linear least‐squares regression analysis and Pearson correlation coefficient**

0.4 < *r* <1 representative significant positive correlation, and the larger the value, the stronger the correlation; -1 < *r* < -0.4 representative significant nagetive correlation, and the smaller the value, the stronger the correlation. Bio1: annual mean temperatures; Bio12: annual mean precipitation; TEM_avg: the mean temperature for 30 days in total before and after the sampling date. Significance levels are denoted as follows: ^*^*P* < 0.05; ^***^*P* < 0.001; ^****^*P* < 0.0001
